# Facilitators and Barriers for a Stepped Care Approach to Promote Return to Work of Employees with Distress: A Multi-perspective Qualitative Study

**DOI:** 10.1007/s10926-025-10301-7

**Published:** 2025-05-16

**Authors:** Hanneke A. M. Lettinga, Sandra H. van Oostrom, Hendrika P. Zijlstra, Johannes R. Anema, Karin I. Proper

**Affiliations:** 1 Center for Nutrition, Prevention and Health Services, National Institute for Public Health and the Environment, Bilthoven, The Netherlands; 2 Department of Public and Occupational Health, Amsterdam UMC, Vrije Universiteit Amsterdam, Amsterdam Public Health Research Institute, Amsterdam, The Netherlands; 3 Zorg van de Zaak Netwerk, Utrecht, The Netherlands

**Keywords:** Sickness absence, Return to work, Distress, Mental health, Stepped care, Rehabilitation

## Abstract

**Purpose:**

A stepped care approach, beginning with a low-intensity e-Health program followed by a high-intensity Participatory Approach led by a return-to-work (RTW) coordinator is a promising intervention to promote RTW of employees on sick leave with distress. As this approach is new, determinants of its successful implementation remain unknown. This study aimed to identify the potential facilitators and barriers for a stepped care approach to promote RTW of employees with distress, from the perspective of employees, employers, and occupational physicians.

**Methods:**

A qualitative study was performed consisting of individual semi-structured interviews with 10 employees, 5 supervisors, and two focus groups with 15 occupational physicians. The verbatim transcripts were inductively thematically coded. The Consolidated Framework for Implementation Research (CFIR) was applied to classify themes within its domains.

**Results:**

Themes were constructed belonging to either the implementation or the content of the stepped care approach, falling within the CFIR domains “innovation,” “outer setting,” “inner setting,” and “characteristics of the individual.” From all three stakeholder groups, identified facilitators were the tailored program, enabling task adjustments, and the RTW coordinator to stimulate a good communicative relationship between employee and supervisor. Barriers identified were the timing of the stepped care approach, integrating the approach in the current RTW system, and low digital skills.

**Conclusion:**

Both facilitators and barriers were found for the implementation of the stepped care approach. This underlines the importance of an adaptive implementation strategy that takes into account workplace dynamics and tailored approaches to support the stakeholder groups’ needs.

**Trial Registration:**

ISRCTN: 90663076. Registered on October 5, 2023.

## Introduction

Common mental disorders (CMDs, such as anxiety, depressed mood, and stress-related disorders) are highly prevalent among the working population, with a recent estimate of around 15% of working age adults experiencing a mental disorder at any point in time [1]. CMDs are one of the leading causes for long-term sickness absence and disability in the workforce [2]. Besides the high impact on the individual, (long-term) sickness absence also places an economic burden on the employer and society. Costs mainly caused by productivity loss due to CMDs was estimated at US$ 1 trillion globally [1]. These challenges also concern the Netherlands, where one in every four (26%) Dutch adults experienced a mental disorder at some point in 2019 [3]. A recent study showed that costs for the employer due to stress-related health problems were above 19 thousand euros [4] per employee.

Before 2010, mental health interventions at the workplace (especially for anxiety and depression) focused primarily on symptom reduction and other clinical outcomes, rather than work-related outcomes [5]. Despite that these interventions are effective on reducing symptoms, the interventions had a negligible effect on the duration of sick leave and time to return to work (RTW) [6–8]. Since the mid-2010s, occupational functioning became an outcome that was more often considered in evaluating workplace mental health interventions [9, 10], and since then there is a growing consensus that the interventions are recommended to not focus on the individual alone, but should also include the workplace [11–13]. Other elements that consistently recur as important factors for RTW in employees with CMDs are (1) to promote RTW early in the absenteeism process [14], (2) having positive RTW expectations of the employee, and (3) to combine individual and workplace approaches [13, 15, 16].

To promote sustainable RTW (defined as full RTW in previous or equal work for at least four consecutive weeks), our research group developed a stepped care approach that integrates both individual- and workplace-directed elements [17]. The stepped care approach is based on components that previously have been found to be effective in intervention studies [15, 16] and starts early in the absenteeism process. The target population for the stepped care approach are employees who experience one or more of non-specific stress symptoms, depressed mood, anxiety, and/or somatization (hereafter referred to as ‘distress’). The stepped care approach starts with a low-intensive e-Health program preferably within two weeks of absenteeism. Based on their responses to questionnaires, employees receive a tailored set of modules relevant to their situation with the focus on developing and reinforcing a positive cognition toward RTW. They can complete the e-Health program independently and at their own pace, with a maximum duration of six weeks. If RTW has not been achieved after six weeks, the e-Health program is followed by a more intensive Participatory Approach (PA). The PA is a stepwise process between the employee and his or her supervisor, under guidance of an independent RTW coordinator. The RTW coordinator first conducts separate obstacle inventories for RTW with the employee and his or her supervisor. In a third, joint meeting, the employee and supervisor work toward consensus on key obstacles for RTW and brainstorm for solutions to enable RTW under the guidance of the RTW coordinator. Based on the solutions, they formulate these in a plan of action which they can implement in practice. After a period of three months, the implementation of solutions is evaluated [17]. The RTW coordinators are trained by the research group in their facilitating role.

At this moment, it is unknown how to best implement the stepped care approach and which key factors to consider beforehand remain unclear. Consequently, it is unknown how to best implement the stepped care approach and which key factors to consider beforehand remain unclear. The RTW process involves various stakeholders, such as the employee, the employer or supervisor, and an occupational health professional. Each of these stakeholders have their own interest and roles [18] during the RTW process, and facilitators and barriers for successfully implementing the proposed stepped care approach can be different based on the perspectives from these different stakeholders. Therefore, the aim of this paper is to identify potential facilitators and barriers for the implementation of a stepped care approach for RTW in employees with distress, based on the perspectives from the important stakeholders in the RTW process in the Netherlands: the employee, supervisor, and occupational physician (OP).

## Methods

### Design

This study used a qualitative design with semi-structured interviews and focus groups to explore stakeholders’ perspectives on the implementation of a stepped care approach for RTW. A reflexive thematic analysis approach [19] was applied to allow for a flexible and iterative interpretation of the data. The study follows the consolidated criteria for reporting qualitative research (COREQ32) [20].

### Sample

A convenience sample of employees, supervisors, and OPs were included in the study. The employees were recruited through a call for participants in the newsletter of the Dutch patient association for mental health problems (MIND). Inclusion criteria were that the employee (1) had experienced an episode of sickness absence with distress (regardless of duration, recent or in the past), (2) returned to work, and (3) was working for an employer at the time (regardless of contract hours). Supervisors were recruited from different organizations: a large occupational health service, a technology consulting firm, and an executive agency of central government. OPs were recruited within the same occupational health service. The inclusion criterion for both supervisors and OPs was that they had experience in guiding one or more employees with distress during sickness absence. The participants recruited for this study had no involvement in the study design, execution, or analysis beyond participation in the interviews. During recruitment, all participants received oral (e.g., before the start of the interview) and written information about the study by means of a flier or information letter.

### Data Collection

Data were collected by online individual semi-structured interviews (using MS Teams) with HL between February 2023 and December 2023 with employees and employers. Focus groups were held in person with the OPs by two interviewers (HL and SOP) in March 2023. Signed informed consent or audiotaped informed consent was obtained. The mean duration of the interviews and focus groups was 1 h.

For all stakeholder groups, two core topics were discussed using multiple questions. The first topic was their experience with sickness absence due to distress and RTW. After the introduction, the interview started with a broad question to invite participants to share their experience with the guidance (either received or given) during sickness absence and RTW. Then, the stepped care approach was introduced with a brief explanation of the goal and content of the approach, which was the same in every stakeholder group. The second topic concerned potential facilitators and barriers (from here: facilitators and barriers) for the implementation of the stepped care approach. The specific question(s) differed between the stakeholder groups. The overall question for employees was as follows: “When you were on sick leave, would you have participated in such a stepped care approach?” with follow-up questions regarding the facilitators, barriers, and preconditions for the stepped care approach. Supervisors were asked about preconditions for implementation of the stepped care approach (e.g., “What are preconditions for a successful implementation of the stepped care approach within the organization?”) and their role as a supervisor (e.g., “the stepped care approach requires active involvement and time investment from the supervisor, in what way does this fit within your work?”). For the OPs, the necessary skills for a RTW coordinator and preconditions for implementing the stepped care approach (e.g., an inventory of skills needed) and facilitators and barriers for successful implementation of the stepped care approach were asked.

### Data Analysis

Most interviews were recorded by the built-in recording feature of MS Teams, while the focus groups and one interview were recorded using an Olympus recording device. All audio files were transcribed verbatim. Any details that could identify participants, such as names and workplaces, were anonymized to ensure confidentiality. Two interviewers, both trained in qualitative research (HL and SOP), coded the transcripts in MAXQDA software. The six steps by Braun and Clarke were followed [19], which entailed (1) getting familiar with the data, (2) initial coding, (3) finding themes inductively, (4) reviewing of themes, (5) defining themes, and (6) reporting the results. During this process, themes were developed bottom-up from the data rather than imposed by predefined categories. Between steps 3 and 4, the updated Consolidated Framework for Implementation Research (CFIR) was used as a theoretical framework to identify the final themes [21, 22]. The CFIR is a widely used framework to guide assessment of contextual determinants of implementation of an innovation, and consists of five major domains (innovation characteristics, outer setting, inner setting, characteristics of the individuals, and the process of implementation) [22].

Given the interpretative nature of qualitative research, reflexivity was considered throughout the analysis. The research team included a psychologist (HL) and a sociologist (SOP), both with expertise in occupational health and RTW interventions. Five interviews (two with employees, two with employers, and one focus group with OPs) were inductively coded separately by the two researchers to develop an initial coding frame. These coding frames were discussed until discrepancies between the frames were resolved, and one joint coding frame remained. This frame was used to code the remaining interviews, while allowing flexibility to adjust the coding frame based on new interviews. After this, the updated CFIR was applied on the themes that were found inductively in the data to define the final themes in terms of the CFIR domains.

## Results

Recruitment resulted in 10 employees (6 female), 5 supervisors (2 female) for interviews, and 15 OPs (7 female) were included and divided over two focus groups. Employees worked across various sectors, including creative and cultural industries, public policy, healthcare and social services, industry, and ICT. All participants in the study had a high educational level. Three supervisors were recruited from the occupational health service, one from a technology consulting firm, and one from an executive agency of the central government.

The identified themes could be classified into two main headings: (1) the implementation of the stepped care approach; and (2) the content of the stepped care approach. The themes were then categorized according to the CFIR domains, aligning with innovation characteristics, outer setting, inner setting, and individual characteristics. Table 1 provides an overview of all themes, CFIR domains, and their corresponding facilitators and barriers.

**Table 1:** Overview of themes, CFIR domains, facilitators, and barriers from the perspective of employees [**E**], supervisors [**S**], and occupational physicians [**O**] for the implementation of the stepped care approach

Theme	CFIR Domain	Facilitators	Barriers
The implementation of the stepped care approach			
Appropriate timing of starting the stepped care approach	Innovation characteristics	Early intervention to maintain structure^**E**^ Early education of stress complaints and lifestyle recommendations^**O**^	Starting too early with the stepped care approach when the employee’s primary need is rest^**E**^
Psychological safety as precondition for implementation	Inner setting	RTW coordinator to promote a psychologically safe environment^**E, S, O**^ RTW coordinator to create awareness among supervisors about possible blind spots^**S**^	
Integrating the stepped care approach within a complex RTW system	Outer setting		Stepped care approach as another health care service on the pile of possibilities and appointments^**E, O**^ Unorganized overview for the OP^**O**^
Digital proficiency and trust to engage with the e-Health program	Individual characteristics		Lack of digital skills in the older generation^**E, O**^ E-Health program content has a high cultural factor, there may be no connection with employees from a non-Western culture^**S, O**^ Reluctancy to share sensitive personal information on a digital platform^**E**^
Content of the stepped care approach			
Tailored information and guidance	Innovation characteristics	Online verified information and instructions tailored to complaints^**E**^ PA as a fully tailored approach to the situation of the employee^**E, S, O**^	
Role clarity and facilitation skills of the RTW coordinator	Innovation characteristics	Independent RTW coordinator to ensure a safe atmosphere for an equal dialogue^**E, S O**^	
A well-established employee–supervisor relationship	Inner setting	PA as a stimulator for a good relationship between employee and supervisor^**E, S, O**^ Provide better care if the supervisor knows what the employee is struggling with^**S**^ Helpful in the intermediary role of the OP^**O**^	
Possibility of adjustment of working conditions	Inner setting	Obstacle inventory in the stepped care approach to clarify what the employee is struggling with, in combination with the prioritization together with the supervisor to determine which obstacles should be addressed^**E, S, O**^	
The lack of knowledge and awareness of medication and its impact on work	Inner setting		The impact antipsychotic medication use has on performance in the workplace^**E, S**^
Prior experience in guidance during sickness absence with distress	Outer setting	A protocolized way to help expressing the needs of the employee and guide the supervisor in the RTW process^**E, S, O**^	
Addressing negative cognitions toward RTW	Individual characteristics	E-Health program to focus on positive cognitions for RTW^**S, O**^	

### The Implementation of the Stepped Care Approach

#### Appropriate Timing of Starting with the Stepped Care Approach (CFIR: Innovation Characteristics)

The stakeholder groups expressed mixed opinions on the timing of introducing the stepped care approach. Some employees and supervisors mentioned that intervening early during sick leave is important to maintain daily structure and bonding with the workplace and viewed the early intervention as a facilitator for implementation. Other employees were hesitant about the early moment of starting with the stepped care approach. Their main concern was the belief that rest is the primary need of an employee who is sick listed with distress, as one employee mentioned:

When you call in sick, you’re also just really tired for a moment, and everything feels like it’s too much. You want to take a step back from your work for a while. And when you’re immediately expected to work on your thoughts, tackle this, look at things differently... I think, at least in the period when I had just called in sick, I thought, phew, I really want to leave it behind for a moment, just take that rest, just nothing for a while.

The OPs mainly named benefits of educating the employees about their stress complaints and providing early guidance on lifestyle recommendations. They noted that, in the current system, their first consult with the employee can take up to 13 weeks, with nurse practitioners providing earlier support. They noted that the e-Health program would already be completed by then and expressed a desire to be more involved in the process.

#### Psychological Safety as Precondition for Implementation (CFIR: Inner Setting)

A psychologically safe working environment was seen as an important precondition for the successful implementation of the stepped care approach by all three stakeholder groups. According to employees, elements of an unsafe social working environment included feeling unable to speak freely, experiencing stigma on distress and job insecurity. One employee described their perspective on the current state of psychological safety in many workplaces:

We say that the employee who isn’t working is the one carrying the problem, and the employer is the one suffering from the problem [of the employee]. The employee is expected to work hard on themselves until they become a “regular” employee again, and maybe then they can return to the company. But it's always an interaction between people, and it could be anything... You can’t really predict what’s going on.

Supervisors mentioned that there is a growing amount of attention for a psychologically safe environment at work and that the PA could help create awareness of possible blind spots.

#### Integrating the Stepped Care Approach Within a Complex RTW System (CFIR: Outer Setting)

In the Netherlands, employees can receive support from multiple services and professionals during sickness absence alongside the guidance from the OP. All three stakeholder groups mentioned that this health care provider system often leads to an unorganized situation, in which many different treatments by different providers could have been mobilized to support the employee. This was a barrier for the stepped care approach, since the employee may already get overloaded with appointments and different plans of action made with the different caretakers. The OPs indicated that they sometimes feel they’re losing track of what is happening with their client, and they expressed the concern that the stepped care approach could possibly cause more confusion than it would be helpful. As one OP mentioned after introducing the PA:

Participatory Approach? It’s a tricky term. I think you really have to be careful not to end up with too many cooks in the kitchen. Because I already notice this sometimes: the employer has one idea, the psychologist has another, then the company doctor, the GP, and even the best friend. And when someone else comes along and says, ‘I think you should do it this way’… (…) You have to be careful with that.

#### Digital Proficiency and Trust to Engage with the e-Health Program (CFIR: Individuals Characteristics Domain)

All stakeholder groups emphasized that a certain level of digital skills is needed to successfully engage in the stepped care approach, specifically with the e-Health program. A lack of these skills may present a barrier for participation. OPs noted that among older workers digital skills are less common, and some employees acknowledged their own lack of digital skills, therefore considered this a barrier for participating in the stepped care approach. In addition, in the situation of sickness absence due to distress, the cognitive resources to learn something new—such as engaging with an unfamiliar platform that requires new or minimally possessed skills—are very limited. OPs and supervisors also mentioned that for employees with a different cultural background or a low educational level, the e-Health program may be inaccessible. Another barrier for engagement with the stepped care approach was concerns about privacy and the supposed impersonal nature of digital tools. Some were reluctant to use a digital platform to share personal information with, and stressed the need for personal contact during sickness absence due to distress, as one employee mentioned:

I’m not... I’m not eager for digital health. I feel like there is a lot being developed, of which I am very afraid it will not going to be used. I think it is very much about... I think it is very much about personal contact which is what you want, and not a module. But I can also imagine that people do want to use it, but I’m not like...‘this could be something’… Whether there is a real need for this.

### Content of the stepped care approach

#### Tailored Information and Guidance (CFIR: Innovation Characteristics)

All stakeholder groups emphasized that access to tailored information and guidance is important in the RTW process, particularly in the early phases of sickness absence. Employees mentioned that they first didn’t know what was happening and searched online for information. Supervisors and OPs also recognized this early need for structured guidance and highlighted that the e-Health program within the stepped care approach could help meet this demand by offering professionally verified information in a structured and accessible way. As one supervisor explained:

These days, you can drive yourself crazy Googling, and you end up finding all sorts of things. So, if you have an app that can provide the right information – something reliable – I think that could be a real addition, because you're probably looking for answers anyway.

#### Role Clarity and Facilitation Skills of the RTW Coordinator (CFIR: Innovation Characteristics)

All stakeholder groups considered the introduction of an external RTW coordinator guiding the structured conversation between the employee and their supervisor in the PA as facilitator, but they stressed the presence of certain conditions, such as a safe culture. Employees and supervisors both emphasized that it is important that the RTW coordinator is an independent actor in order to stimulate an equal dialogue. For the RTW coordinator to effectively facilitate an equal dialogue, it is important to establish clear roles and preconditions at the start of the PA. This ensures that all parties understand the framework within which discussions take place, creating a structured and safe environment where both the employee and supervisor can openly express their perspectives. Supervisors acknowledged this, as one supervisor explained after the PA was introduced:

If I were confronted with that, I would start thinking about: what does that mean for my position? And how safe is that for me? And I can imagine that from both the employee’s perspective and the supervisor’s perspective. Both are in separate situations and... So for that safety, I would want to pay attention to that in some way. I’d want to have that discussed. So, in that meeting – the introductory meeting with the person guiding the process – I’d want to address that aspect.

The OPs often noticed a hierarchical relationship between employee and supervisor. Some mentioned that sometimes an employee is too afraid to share some of their struggles with their supervisor, because of expected consequences, accentuating the importance of preconditions for an equal dialogue which can be facilitated by the RTW coordinator.

#### A Well-Established Employee–Supervisor Relationship (CFIR: Inner Setting)

All stakeholder groups emphasized that a strong relationship between the employee and supervisor is beneficial for RTW and saw opportunities within the stepped care approach to support and strengthen this relationship. The PA was specifically mentioned as a tool to facilitate structured conversations between both parties. Supervisors noted that open and continuous communication with employees helped them offer better support, while OPs emphasized that early and clear dialogue between employees and supervisors allowed them to perform their intermediary role more effectively. All three stakeholder groups mentioned that the independent RTW coordinator could facilitate this open communication structure by facilitating constructive conversations. After asking an employee whether he would have (hypothetically) participated in the PA during his sickness absence, he responded:

It really depends a lot on the relationship with your supervisor, because with that last lady, I didn’t necessarily need all of this. I might have participated, but together, we worked it out well. But I do think it can be helpful to have someone independent. You see, the supervisor... Look, someone with a broken leg is very clear: it takes six weeks, and then they can go back. But all those psychological issues, many supervisors find that scary or they don’t know anything about it. Or they still have to manage an entire department. They’re just dealing with it on the side.

#### Possibility of Adjustment of Working Conditions (CFIR: Inner Setting)

The stepped care approach, in which a task analysis and obstacle inventory is performed, was a facilitator for RTW as it provides insight whether or not specific tasks are (currently) an obstacle for RTW. Being able to adjust the work content, work pace or other working conditions were mentioned as important factors in the RTW process by the employees, supervisors, and OPs. In general, supervisors and OPs mentioned that examining whether the employee still fits in their current job is part of the reintegration process, as sometimes employees with recurrent sickness absence due to distress may not have a great fit (anymore) with the organization or team they’re working in. They also considered that the e-Health program, which is aimed at the cognition toward RTW, could serve as a facilitator in reflecting on causes of the sickness absence, as one OP mentioned:

I think it would be really valuable if the e-health could contribute to gaining insight into those vulnerable undercurrents. That people feel safe enough to gain that insight, that this could also be a reason why things aren’t progressing. It’s not because they’re psychologically vulnerable or because they don’t have the right psychologist, but it could simply be that something like work is just going badly or something like that. Because if you have that insight after someone has gone through such an app, yeah, you’re going to approach things very differently than if, after week 30, it comes out that someone doesn’t feel safe enough to say that.

#### The Lack of Knowledge and Awareness of Medication and Its Impact on Work (CFIR: Inner Setting)

Some employees discussed the medication prescribed for their condition or symptoms, noting a lack of information about its potential effects on their work performance. They also experienced insufficient guidance on what to expect when returning to work while using medication. One supervisor recalled an employee returning from sickness absence, stating: “…*Well, he was there, but he was still under his medication…*.” Both the employees and this particular supervisor did not feel adequately prepared for the impact of the medication use on their work performance, as it caused severe side effects, such as fatigue and a decline in fine motor skills. At the same time, privacy regulations protect employees from having to disclose private medical details (including medication use) to their employer or colleagues. The employees in this situation did not want to share these details with their employer, resulting in that the employers were unaware that medication side effects could affect the employee’s functioning. This led to misunderstandings: employees, unwilling to share private medical details, struggled with unexpected performance difficulties, while employers, assuming full recovery, expected them to resume work without adjustments. One employee, who returned to work after hospitalization, described the situation as follows:

For instance, I took an antidepressant for a while, which caused my fine motor skills to deteriorate. That was really terrible: I could simply no longer perform actions that I could ‘normally’ do. I could no longer grasp small pieces. That was really terrible. (…). That was really bad. I thought I would lose my job if I continue taking this medication.

#### Prior Experience in Guidance During Sickness Absence with Distress (CFIR: Outer Setting)

The employees noted that when they encountered an OP or supervisor who had some prior experience with guiding sick-listed employees with distress, they felt like they had better support during their RTW process. At the same time, supervisors indicated a lack of knowledge to properly guide their employees through the sickness absence and RTW process—as they are experts on the content of their field and not necessarily on guiding employees with distress. As one supervisor mentioned:

As a supervisor, you’re usually rather in the content of the work anyway, and to ensure progression on that matter… Especially if you consider the part of counseling people who have had mental health problems as a result of work or whatever. That’s actually not what you're trained for. I’m a mechanical engineer and I’m not a psychologist, so to speak. So I always find that... You get that part of the job automatically – and you develop it a little bit, but you’re not necessarily the expert in that, I think.

A protocolized way to help the employees putting their symptoms into perspective using the e-Health program and helping to express their needs through the PA was considered as facilitator for the stepped care approach.

#### Addressing Negative Cognitions Toward RTW (CFIR: Individuals Characteristics)

All stakeholder groups recognized that expectations and cognitive attitude toward RTW play a crucial role in the reintegration process. Some employees expressed eagerness to RTW as soon as possible, stating that staying at home left them with less structure and a sense of non-contribution to society. For others however, thinking about RTW served as a trigger that, in their experience, worsened their symptoms. Supervisors encountered cases where employees became “stuck in their own thinking pattern,” leading to supposed delayed RTW. OPs recognized these experiences and contradictory beliefs among employees and considered there is also an important role for the social network of the employees in forming these beliefs. As one OP described,

I always get really irritated by the therapists, and often not really a psychologist or psychiatrist saying something, but usually more the practice assistants and even more so the alternative people who say things like: “Well, you should count on at least six to nine months,” or “Yeah, don’t even think about it for the first year…” That really clashes... It’s so hard to change that expectation once it’s been set, while you think, this didn’t have to happen at all. People then also have a kind of defensive instinct to say: “Yeah, but it’s going to take a year.” But I think, I’m not the one who’s going to put you back in there. I want to have a conversation with you. But they say: “No, it’s not the time yet because I still have a year.”

In this respect, supervisors and OPs responded positive to the e-Health program, which focuses on these cognitions toward RTW could help in creating awareness of one’s own thoughts and processes.

## Discussion

The current study explored potential facilitators and barriers for the implementation of a stepped care approach for RTW of employees with distress. Three stakeholder groups (employees, supervisors, and OPs) shared their perspectives on factors that could influence implementation. Their input could broadly be classified into two directions: facilitators and barriers for the implementation and facilitators and barriers for the content of the stepped care approach. For the implementation, key themes were timing of the intervention, psychological safety in the workplace as a precondition, integrating the stepped care approach within the existing RTW system and digital proficiency and trust to engage with the e-Health program. Concerning the content of the stepped care approach, key themes were tailored information and guidance, role clarity of the RTW coordinator, workplace relationships, cognitions toward RTW, prior experience in guidance during sickness absence with distress, possibility of adjustment of working conditions, and medication awareness. All themes were categorized within the CFIR framework, aligning with the domains of innovation characteristics, outer setting, inner setting, and individual characteristics.

Within this framework, the themes that emerged under the implementation of the stepped care approach reflected the current state of either the individual or the organization, whereas the content of the stepped care approach reflected its potential to facilitate a change at either level. For example, while early intervention is often recommended to prevent long-term absenteeism [14], some employees mentioned that starting early with the intervention might be overwhelming, while others were surprised that the PA would only be introduced after six weeks. This variation in perspectives highlights that the timing of starting with the approach (an implementation factor) reflects the individual’s state of readiness to engage in RTW efforts. However, it may also indicate a negative attitude toward RTW, a content factor. The e-Health program, introduced early in the absenteeism process, aims to address negative attitudes toward RTW and encourages a more constructive RTW mindset, while allowing employees to engage with it at their own pace. This aligns with the current guideline for RTW with mental health problems of the Netherlands Society of Occupational Medicine (NVAB), which recommends a process-contingent rather than a time-contingent approach, emphasizing the importance of tailoring interventions to an employee’s progress in recovery rather than adhering to a fixed timeline.

The same dynamic holds for the introduction of an independent RTW coordinator during the PA. The coordinator’s role in creating a safe environment for open, equal dialogue between the supervisor and employee, and guiding them throughout the RTW process, was anticipated as a facilitator for implementation by addressing critical relational and process factors. Employees identified a psychologically safe working environment as a precondition for the successful implementation of the stepped care approach, yet the structured facilitation provided by the RTW coordinator may also actively contribute to creating this safety. The structured meetings—designed to reach consensus on key barriers and solutions—were well received. These findings build on earlier research indicating that a facilitated dialogue led by an RTW coordinator supports the RTW process itself [23]. In a study that evaluated barriers and facilitators for RTW according to employees with different lengths of sickness absence [24], overlapping themes were found with the current study, highlighting the essential role of adequate work adjustments, the relationship with the supervisor, and professional guidance as significant RTW factors. These findings underscore an important implication: for the RTW coordinators to be effective, they must be equipped with strong facilitation skills and the ability to set the right conditions at the start of the meetings, ensuring that both employees and supervisors feel supported in engaging in a constructive dialogue.

In the current study, supervisors and OPs emphasized that, in some cases, an employee’s recurrent sickness absence may stem from no longer fitting within their current job or organization. OPs saw a potential benefit within the e-Health program in providing structured self-reflection on work-related stressors and job expectations, helping employees develop insights into whether their current job still aligns with their needs​. While the e-Health component may support individual reflection, addressing job fit concerns requires a structured dialogue between the employees and their employer. The PA presents an opportunity to facilitate this discussion, allowing employees to explore potential adjustments within their current role or consider alternative reintegration pathways, either within or outside the organization. These findings raise an important consideration for the scope of the current stepped care approach, which is primarily designed to facilitate RTW at the same employer and does not have a focus on job transitions. However, this may not be a major limitation, as the intervention is deployed early in the absenteeism process. Previous research showed that job transitions are more often necessary in cases of long-term sickness absence, where changes in work tasks over time can lead to lower job satisfaction [24]. By intervening at an early stage, the stepped care approach may help prevent long-term sickness absence and increase the likelihood of successful return to work within the same organization. Based on these findings, it is important that if the stepped care approach proves to be effective and its implementation is expanded to include employees with recurrent or long-term sickness absence, its design may need to be expanded to include job transition support. The e-Health program currently tailors its modules to the individual’s situation, which presents an opportunity to include an additional module on job transition for employees with recurrent or long-term sickness absence, where reintegration within the same organization may not be feasible.

Another potential barrier mentioned by the employees and one supervisor concerns the side effects of medication and the influence it has on the employees’ work functioning. The employees who had medication prescribed during their sickness absence all mentioned that they suffered from the side effects in the workplace which led to misunderstanding by their supervisor or colleagues. Although employees are not required to disclose medical information to their employer, they may choose to discuss medication-related concerns privately with the RTW coordinator. The PA meetings could provide a structured but confidential setting where the RTW coordinator helps the employee identify work tasks that may be affected by medication side effects. In the three-way meeting, these obstacles can then be addressed in a solution-focused way, for example, by discussing temporary work adjustments in tasks, schedules, or workload without requiring disclosure of the specific medication. Despite there being a wide range of pharmacological interventions for distress, the effects of taking medicine on work performance have not been well studied. Actually, these interventions focus on symptom reduction, while previous research already showed that symptom-focused interventions have limited impact on work outcome measures [6, 8–10, 25]. This illustrates that the impact of medication on work functioning should not be overlooked, as it could be an important barrier for RTW and therefore must not be ignored during the PA.

Finally, there were two potential barriers for implementation that emerged from the stakeholder discussions: digital accessibility and integration of the stepped care approach within the complex RTW system. All stakeholder groups expressed concerns about the level of digital skills necessary to engage with the e-Health program. Adaptations have been made to enhance accessibility for independent users by following the Common European Framework of Reference for Languages [CEFR] standards; [26] and incorporating additional educational clips to reduce the need for extensive reading. However, future adaptations should also consider expanding language options and incorporating cultural aspects to better support diverse user groups. Another barrier that was mentioned by the stakeholder groups is the variety of health services that already exist, and how the stepped care approach differentiates from existing interventions. This implies a clear overview of the advantages and disadvantages of the stepped care approach, as well as its distinct role within the broader landscape of health services. If the stepped care approach proves to be effective, future research could focus on comparing the (cost-)effectiveness of the stepped care approach with other health services already in place.

### Methodological Considerations

The current study entails some strengths and limitations. The multi-perspective insights from the three important stakeholder groups, and their integration, provides a broad overall view of the potential facilitators and barriers for the implementation of the stepped care approach.

Although the channels used had significant reach, the resulting convenience sample was relatively small and lacked diversity in educational background, as all participants were highly educated. This limitation must be considered when interpreting the results, as the sample may not fully represent the broader population. However, supervisors and occupational physicians provided insights into employees with more diverse educational backgrounds, mitigating this issue to some extent. While interviews continued until data saturation was reached, a purposive sampling strategy would have likely produced a larger, more heterogeneous sample, thereby enhancing the study’s generalizability.

The interviews aimed to identify potential facilitators and barriers for implementing the stepped care approach, and the CFIR was used as a theoretical framework to organize and present the findings. However, the CFIR was applied post-interview, reflecting a more explorative and inductive approach to data collection. Had the CFIR been used beforehand to guide the interviews, it may have allowed for a deeper exploration of its specific subdomains. Nonetheless, the open-ended nature of the interviews and thematic analysis following Braun & Clarke ensured that the researchers were not tied by the framework’s structure, allowing more open conversations. This inductive approach likely captured additional insights, such as the impact of medication on RTW, which may have been missed with a more deductive method.

The backgrounds and experiences of the two researchers may have influenced the thematic coding, interpretation, and framework application. As one of the researchers (HL) was involved in the design of the stepped care approach, this provided a valuable insight into how the intervention could accommodate potential barriers. However, prior involvement could also indicate a favorable bias toward the feasibility of the approach. To mitigate this, themes were developed inductively together with another researcher, who was not part of the design of the stepped care approach. This iterative approach, involving continuous discussion and comparison of codes, allowed for sense checking of interpretations, ensuring that themes were shaped by the data rather than researcher expectations.

## Conclusion

This study presents potential facilitators and barriers for implementing a stepped care approach for RTW in employees with distress, based on perspectives from employees, supervisors, and OPs. Facilitators included the tailored nature of the approach, the RTW coordinator’s role in facilitating structured communication, and its potential to accommodate job fit concerns and medication use. Future research could explore whether these topics should be explicitly integrated into the intervention design. Barriers included timing concerns, digital proficiency requirements, and challenges in integrating the approach within the existing RTW system. To improve accessibility and adoption, further research should examine (cost-)effectiveness compared to other occupational health services and to address digital literacy barriers.

These findings underscore the need for an adaptive implementation strategy that considers individual and organizational readiness, workplace psychological safety, and accessibility challenges. Taken together, the stepped care approach has the potential to support sustainable RTW in employees with distress.

## Data Availability

The datasets generated during and/or analyzed during the current study are available from the corresponding author on reasonable request.
